# Spatial epidemiology of porcine reproductive and respiratory syndrome in Thailand

**DOI:** 10.1186/s12917-014-0174-y

**Published:** 2014-08-05

**Authors:** Weerapong Thanapongtharm, Catherine Linard, Nutavadee Pamaranon, Sarayuth Kawkalong, Tanom Noimoh, Karoon Chanachai, Tippawon Parakgamawongsa, Marius Gilbert

**Affiliations:** 1 Department of Livestock Development (DLD), Ratchatewi, Bangkok, Thailand; 2 Biological Control and Spatial Ecology, University of Brussels, Brussels, Belgium; 3 Fonds National de la Recherche Scientifique (FNRS), University of Brussels, Brussels, Belgium; 4 National Institute of Animal Health, Ladyao, Chatuchak, Bangkok, Thailand; 5 Field Epidemiology Training Program for Veterinarian (FETPV), Department of Livestock Development, Ratchatewi, Bangkok, Thailand

**Keywords:** Spatial epidemiology, PRRS, Autologistic multiple regression, BRT, Breeding sows, Thailand

## Abstract

**Background:**

Porcine reproductive and respiratory syndrome (PRRS) has become a worldwide endemic disease of pigs. In 2006, an atypical and more virulent PRRS (HP-PRRS) emerged in China and spread to many countries, including Thailand. This study aimed to provide a first description of the spatio-temporal pattern of PRRS in Thailand and to quantify the statistical relationship between the presence of PRRS at the sub-district level and a set of risk factors. This should provide a basis for improving disease surveillance and control of PRRS in Thailand.

**Results:**

Spatial scan statistics were used to detect clusters of outbreaks and allowed the identification of six spatial clusters covering 15 provinces of Thailand. Two modeling approaches were used to relate the presence or absence of PRRS outbreaks at the sub-district level to demographic characteristics of pig farming and other epidemiological spatial variables: autologistic multiple regressions and boosted regression trees (BRT). The variables showing a statistically significant association with PRRS presence in the autologistic multiple regression model were the sub-district human population and number of farms with breeding sows. The predictive power of the model, as measured by the area under the curve (AUC) of the receiver operating characteristics (ROC) plots was moderate. BRT models had higher goodness of fit the metrics and identified the sub-district human population and density of farms with breeding sows as important predictor variables.

**Conclusions:**

The results indicated that farms with breeding sows may be an important group for targeted surveillance and control. However, these findings obtained at the sub-district level should be complemented by farm-level epidemiological investigations in order to obtain a more comprehensive view of the factors affecting PRRS presence. In this study, the outbreaks of PRRS could not be differentiated from the potential novel HP-PPRS form, which was recently discovered in the country.

## Background

Porcine reproductive and respiratory syndrome (PRRS) is a pig disease that emerged in the last part of the twentieth century and spread through pig production systems. The disease was first recognized in the United States in 1987 and in Europe in 1990 [1]. Today, the disease has become endemic in many countries throughout the world following an epidemic phase [1]. The PRRS viruses (PRRSV) mainly cause reproductive failure in sows and respiratory disease in piglets and therefore significantly impact the productivity of affected pig production systems [2]. The viruses can directly transmit through placenta and body fluids of infected animals as well as through contaminated fomites, vectors and aerosols [3]. So far, the viruses have not been found in other host species in natural conditions [1]. In experiment conditions, some avian species, mallard ducks in particular, have been found to be able to harbor and shed the viruses for several days after challenged [4]. Following infection, most pigs clear the virus in 3–4 months *in vivo* but some pigs have been reported to remain carriers of the virus for several months [5].

Previous epidemiological studies on PRRS herd-to-herd transmission risk identified the introduction of infected pigs or semen into the herds as an important risk factor, with subclinical and persistently infected pigs playing an important role in the spread of the disease [6]. Several other farm-level risk factors have been previously reported, including the purchase of animals from herds with PRRSV, the use of semen for artificial insemination from infected boars, high herd size [7],[8], poor farm management practices (e.g. not isolating animals after purchase [7]), or the use of modified-live vaccines [8],[9]. At a higher geographical level, infections from neighboring farm by aerosol spread [8], and high densities of pig farms in the immediate area have also been recognized as risk factors [9].

In April 2006, an atypical form of PRRS was reported in China, Jiangxi province [10]. The new strain caused high and continuous fever, red discolorations of the skin, blue ears in the late phase of the disease, and high mortality rate in all ages of pigs. The virus was subsequently identified as highly pathogenic porcine reproductive and respiratory syndrome (HP-PRRS) characterized by a 30 amino-acid deletion in the nsp2-encoding region of the PRRSV [10],[11]. The PRRSV genome is approximately 15 kb in length and composed of 9 open reading frames (ORFs), of which ORF1a and ORF1b encode 12 non-structural proteins (nsp). The nsp2-encoding region is genetically the most variable area and crucial for viral replication due to its protease activity [12]. When compared to the typical strain, the new one showed a higher tissue tropism *in vivo*, which may contribute to its higher virulence [13]. Since 2007, this new HP-PRRS has been reported in many other countries throughout Asia [14]–[17].

In Thailand, the mild form of PRRSV was first isolated in 1996 from piglets with chronic respiratory distress and the virus was subsequently identified as the US genotype [18]. Since this first documented occurrence, all PRRSV strains isolated in Thailand have been identified as belonging to the EU and US genotypes [12],[19]. The import of pigs or semen from European and North American countries [19], or the smuggling or unauthorized use of modified-live vaccine [20] are possible pathways of entry into the country. There was no evidence of introduction of the new HP-PRRS in Thailand [12],[21] until its first report in pig farms located in the northeast of Thailand in 2010 [16]. Since then, the import of PRRSV-positive animals in the country was banned by the Thai authorities [21], but it is likely that the virus may have been introduced to Thailand through the illegal imports of infected animals [16].

In order to deal with PRRS outbreaks, animal quarantine, movement control, disinfection of infected premises/establishments, treatment of affected animals, and surveillance in and out the affected area were implemented and coordinated by the Department of Livestock Development (DLD) staff [22]. In addition, a public awareness campaign was launched to disseminate information on prevention good practice measures and identification of early symptoms. The campaign targeted pig farmers and aimed to provide them with detailed information on PRRS. Moreover, the campaign encouraged them to enhance their biosecurity level and to apply basic precautionary management practices such as using disinfectant before entering farms and quarantining newly introduced pigs, among other measures [22].

The objective of this study was twofold. First, we aimed to provide a first description of the spatio-temporal pattern of all PRRS cases in Thailand in 2010. Second, by assembling a series of variables quantifying the distribution of different pig types in the country and anthropogenic factors, we also aimed to carry out a first investigation of the risk factors most strongly associated with all PRRS occurrences, possibly including HP-PRRS in Thailand.

## Results

**Figure 1:**
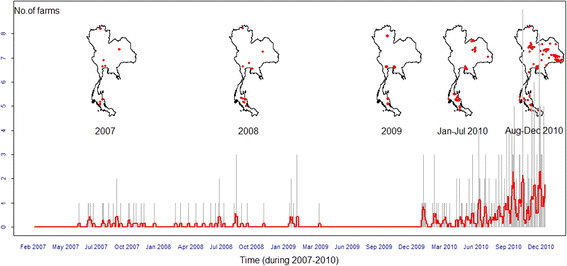
**Spatio-temporal distribution of PRRS in Thailand during 2007 to 2010.** The epidemic curve shows the temporal distribution of PRRS occurrence in Thailand from 2007 to 2010. The grey lines represent the daily number of positive farms, and the red line represents the weekly average number of positive farms. The maps show the spatial distribution of PRRS in Thailand in 2007, 2008, 2009, January to July 2010, and August to December 2010, respectively.

The temporal pattern of PRRS in Thailand (Figure 1) demonstrated only sporadic occurrences in 2007, 2008, and 2009. In 2010, the outbreaks from January to July 2010 were more frequent than in the previous 3 years, followed by a gradual increase from August to December 2010. A simple trend analysis indicated that the weekly occurrence tended to increase over time in the period of interest (linear regression, F_1,363_ = 102.7, *p* < 0.001). The date that maximized the F-statistic of the linear regression including the day number and the period as a dummy variable was the 1^st^ of August 2010 (F_2,362_ = 19.6, *p* < 0.001). During the first period (January - July 2010) and second period (August to December 2010), there was 0.4% (n = 33) and 1.1% (n = 82) of sub-districts reporting at least one outbreak, respectively.

**Figure 2:**
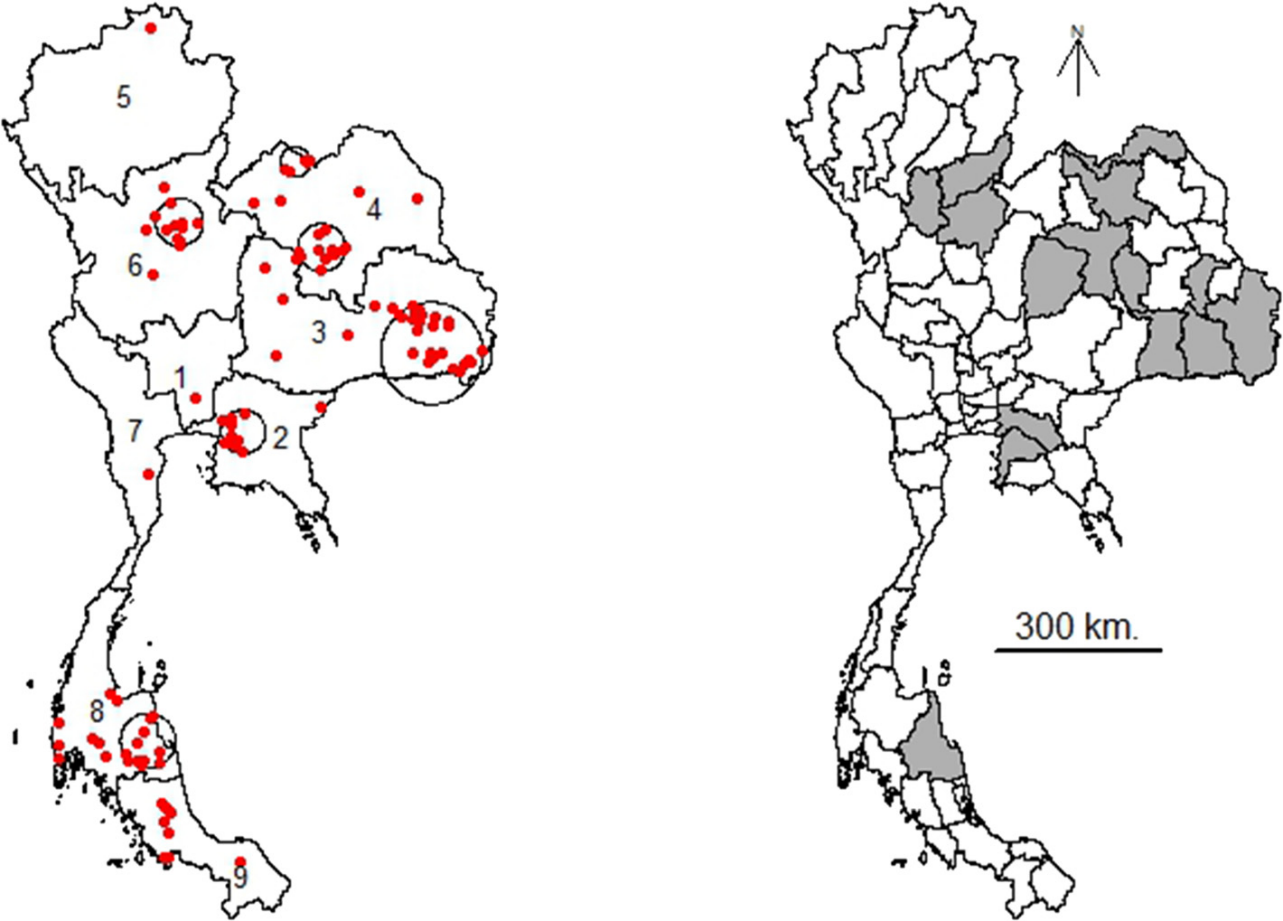
**Outbreak clusters identified by the spatial cluster analysis.** The six clusters of PRRS in Thailand in 2010 identified using the spatial scan statistic (left map) were located in the South-East of region 3 (second period), the West of region 2 (first and second periods), the South-East of region 8 (first and second periods), the South-West of region 4 (first and second periods), the North-East of region 6 (second period), and the North of region 4 (first period). The provinces where these clusters occurred in 2010 are indicated in grey (right map).

The cluster analysis identified six spatial clusters of PRRS outbreaks in 15 provinces of Thailand across the 2 periods in 2010 (Figure 2). The primary cluster occurring in the second period and was located in the South-East of region 3, followed by 5 secondary clusters located in 4 regions : the West of region 2 (in the first and second periods), the South-East of region 8 (in the first and second periods), the South-West of region 4 (in the first and second periods), the North-East of region 6 (in the second period), and the North of region 4 (in the first period), respectively. The details of the spatial clusters are provided in Table 1.

**Table 1:** Details of the spatial clusters of PRRS in Thailand in 2010

Cluster (period*)	**Location**	**Area (km2)**	**Log likelihood ratio**	**P-value (999)**	**Relative risk**	**Prevalence**
1 (2^nd^)	The South-East of region 3	91.36	30.93	0.001	8.73	0.068
2 (1^st^, 2^nd^)	The West of region 2	39.86	20.47	0.001	16.59/8.07	0.061/0.082
3 (1^st^, 2^nd^)	The South-East of region 8	49.48	19.39	0.001	24.68/1.65	0.081/0.018
4 (1^st^, 2^nd^)	The South-West of region 4	43.09	17.22	0.001	15.04/6.32	0.056/0.064
5 (2^nd^)	The North-East of region 6	42.95	16.73	0.004	13.41	0.13
6 (1^st^)	The North of region 4	26.98	14.42	0.012	47.12	0.18

*The 1^st^ period was during January to July 2010 and the 2^nd^ period was during August to December 2010.

Characteristics of the six spatial clusters of PRRS in Thailand in 2010 identified using the spatial scan statistic with multivariate scan test (January to July 2010 and August to December 2010), cluster size of a maximum of 20% of observations, 999 iterations, and Bernoulli model.

**Figure 3:**
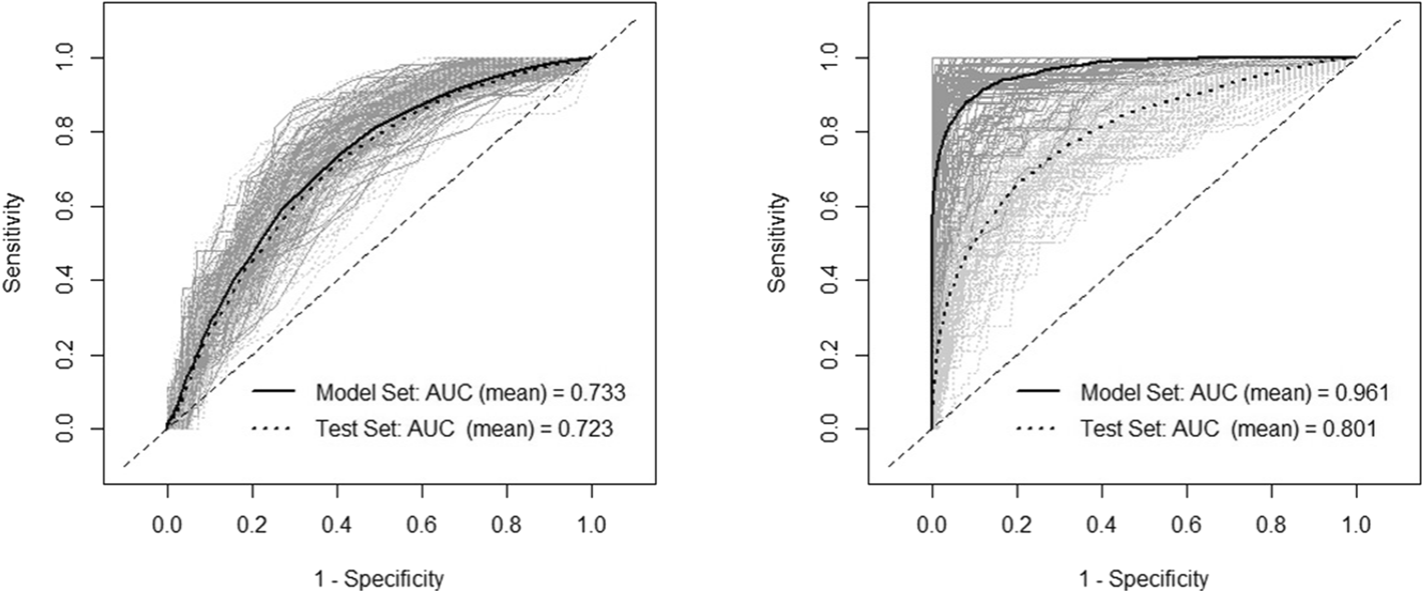
**ROC curves of the predictive power of the models.** ROC curves of the multivariate logistic regression model (left) and the boosted regression tree model (right) on presence/absence of PRRS, from August to December 2010 at the sub-district level. The grey lines represent curves from individual bootstraps, and the thick black lines represent the average AUC curve estimated on the training set (continuous line) and test set (dotted line).

The risk factors found significant at the alpha level of 0.1 in the univariate logistic regression analysis were (i) the number of farms with fattening pigs (*p-value* = 0.062), (ii) the number of farrow-to-finish farms (farms which include breeding, producing piglets and fattening pigs) (*p-value* = 0.061), (iii) the human population (*p-value* = 0.005), (iv) the number of farms with breeding sows (*p-value* = 0.016), (v) the density of farms with breeding sows (*p-value = 0.024*), (vi) the number of farms with breeding piglets (*p-value* = 0.031), and (vii) the density of farms with breeding piglets (*p-value* = 0.071). In the multivariate logistic regression model (Model I), the only variables found simultaneously significant were the human population and the number of farms with breeding sows (Table 2). Both variables were positively associated with the presence of the virus, with the change in log-likelihood upon removal of the number of farms with breeding sows (7.20) being slightly greater than that of the human population (6.28). The predictive power of the model, as quantified by the area under the curve (AUC) of the receiver operating characteristics (ROC) plots (as showed in Figure 3), was moderate (the mean AUC was 0.733 of the model set and 0.723 of the test set).

**Table 2:** Results of the multivariate logistic regression model

**Variables**	**Mean coefficient**	Mean SE*	**Mean odds ratio (OR)**	
			**Adjusted odds ratio**	**95% confidence interval**
Constant	−2.611	0.428		
No. of human population	0.479	0.253	1.669**	1.024 -2.779
No. of farms having breeding sows	1.750 × 10-2	1.823 × 10-2	1.016	1.004-1.029
Autoregressive term	2.438	0.521		

*SE stands for standard error.

**The odds of a sub-district being PRRS positive was increased by a factor of 1.669 (95% CI 1.024 to 2.779) for every 10,000 unit increases in the number of human population in a sub-district.

Results of the multivariate logistic regression model (Model I) with 100 bootstraps applied for analyzing PRRS presence/absence at the sub-district level in Thailand.

**Figure 4:**
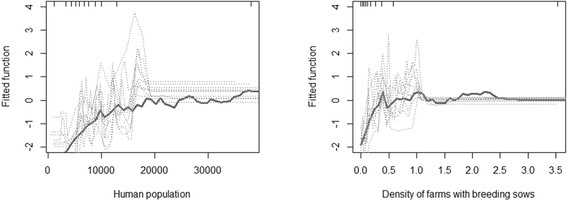
**Fitted function predicted by the BRT.** Partial dependence plots show the effect of a predictive variable on the response after accounting for the average effects of all other variables in the model; fitted function for the number of human population (left) and the density of farms with breeding sows (right)

The BRT model (Model II) was run with a tree complexity of 3, a learning rate of 0.003 and a bag fraction of 0.5. The combination of two variables yielded the maximum predictive power with a mean AUC of 0.961 on the model set and 0.801 on the test set (Figure 3). The two variables selected by our forward entry procedure were the human population and the density of farms with breeding sows, with an average respective relative contribution of 51.64 and 48.36, respectively (Table 3). The fitted function of the BRT model shows the effect of the predictive variable on the predicted response [23], with both variables showing a positive association with the fitted function (Figure 4).

**Table 3:** Results of boosted regression trees

**Variables**	Rel. Con.* (mean)	**Rel.Con. (SD)**	Mean of AUC** (range)	
			**Model set**	**Test set**
No. of human population	51.64	5.44	0.961 (0.838-1.000)	0.801 (0.692-0.908)
Density of farms having breeding sows	48.36	5.44		

*Relative contribution.

**Area under the curve (AUC) of the receiver operating characteristics (ROC) plots.

Results of boosted regression trees (Model II) with 100 bootstraps applied to model PRRS presence/absence at the sub-district level in Thailand.

When crosschecking the significant variables from both models, it was found that both risk factors identified by the forward-entry BRT model were significantly associated with disease occurrence when analyzed using the multivariate logistic multiple regression, but yielded a lower AUC than the variable selected directly by the multivariate logistic regression. Conversely, variables selected by the backward removal multivariate logistic regression were also providing good predictions when used in the BRT, but here too, with a lower AUC compared to the variables selected by the BRT approach.

**Figure 5:**
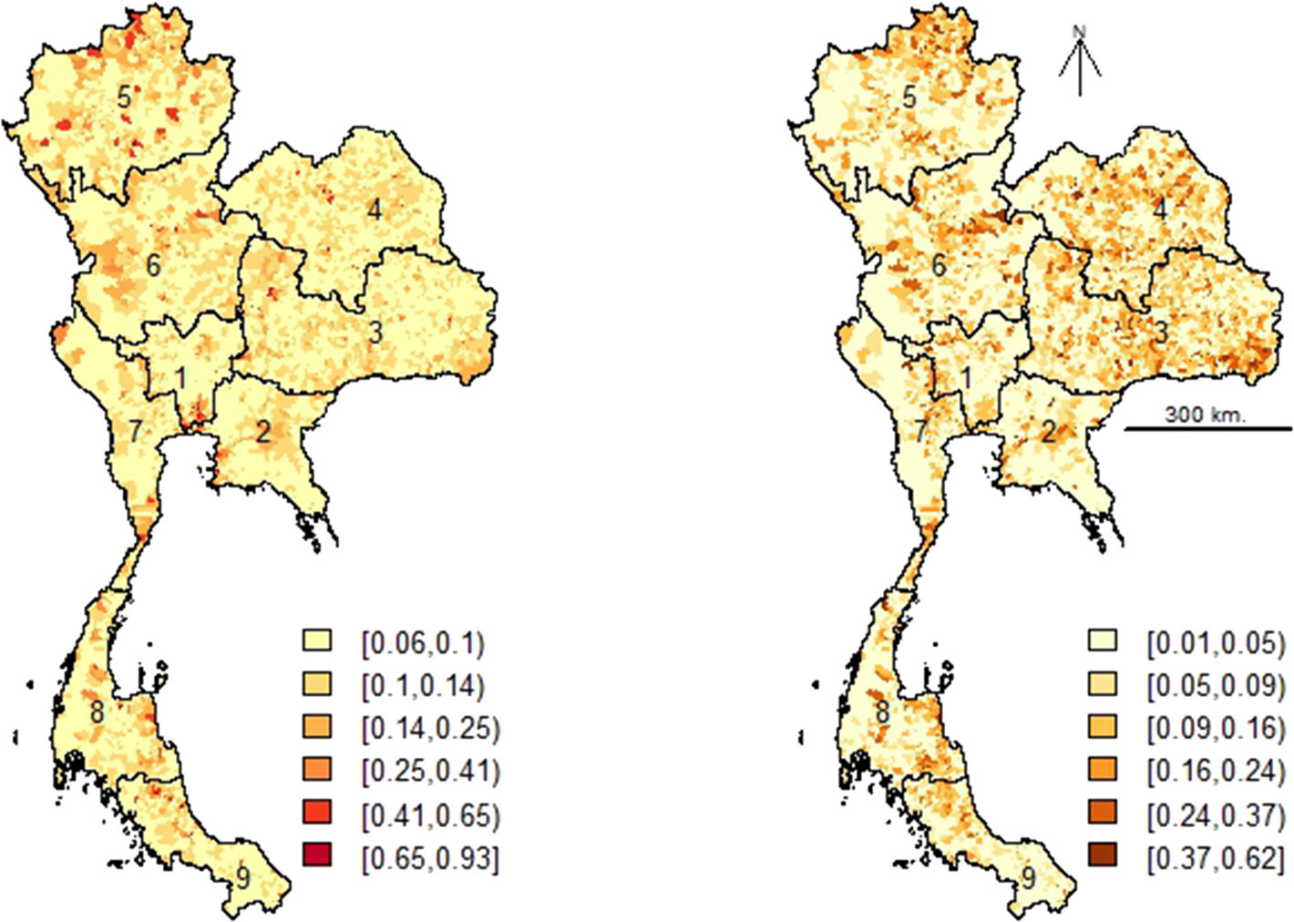
**Predicted PRRS probability of presence in Thailand during August to December 2010.** PRRS risk map predicted by the multivariate logistic regression model (left) and by the BRT model (right).

The probability of PRRS presence predicted by the two models (Model I and Model II) was mapped for all sub-districts (Figure 5). Isolated high risk areas were distributed in several parts of the country in both maps with a higher frequency in the Northeast (regions 3 and 4), the North (regions 5 and 6) and the South (region 8 and along the border of regions 8 and 9). The map predicted by Model II, with higher predictive power, we allows identifying 9 areas with higher risk than the rest of the country: (i) the Northern area of region 5; (ii) the Northeastern areas of region 6; (iii) the Western area of region 6; (iv) the Northern area of region 4; (v) the Southern areas of region 4; (vi) the Northwestern areas of region 3; (vii) the Southeastern area of region 3; (viii) the central area of region 2; and (ix) the border area between regions 8 and 9.

## Discussion

The year 2010 may have marked a change in the epidemiology of PPRS in Thailand, with the first notification of HP-PRRS in the country. A change is apparent, both in the spatial distribution of PRRS and in the average number of cases reported daily. Three main hypotheses may explain this change. First, the common PRRS may have started spreading more extensively than before. Given that the disease has been known to be in the country, and quite widespread, for a reasonable amount of time, it seems unlikely that it would have suddenly spread to new areas for no apparent reason (e.g. changes in production systems, trading patterns, etc.). Second, the new and more pathogenic HP-PRRS variant could have started spreading in the country [16], resulting in more distinct clinical symptoms that made the disease more apparent, resulting in an increase in reported cases. Third, the first detection of HP-PRRS in Thailand may have triggered an increased awareness of farmers and veterinary officers to PRRS symptoms, which may also have contributed to a higher number of reported cases. It is difficult to formally differentiate these hypotheses in the absence of empirical data making a virological distinction between infections caused by PRRS and HP-PRRS viruses. However, the spatial and temporal patterns support that the period ranging from August to December should be analysed separately, since one can assume a different level of reporting during the first period.

Both statistical approaches used to explain the distribution of PRRS identified human population as an important risk factor associated with the occurrences of PRRS at the sub-district level. Human population is often found as a risk factor in livestock infectious diseases, such as for example highly pathogenic avian influenza [24]–[26], and this can reflect two mechanisms. Transmission can be supported by farm-related activities such as transport of outputs (pigs), or inputs (feed, piglets) through contaminated equipment, vehicles, boots, and others [1],[3],[27], allowing the disease to be introduced to other farms (between farms) or other pigs (within farm). One can intuitively assume that an isolated farm in a sub-district with a low human population could have a lower number of possibilities of transmission through those activities than farms located in densely populated areas. However, the positive statistical association between PRRS and human population may also simply result from a reporting bias, with farms located in the most populated sub-districts being more likely to report disease problems, either because farmers were better informed through information campaigns, or because significant mortality would be more easily witnessed by other farmers.

Both statistical modeling approaches also identified an association between the number of farms with breeding sows, either expressed in absolute terms (logistic regression) or as density (BRT). Whilst this specific factor has not yet been reported elsewhere, the most similar result was obtained by Weigel *et al*. [7], who found an increased risk of PRRS diagnosis associated with a higher number of sows. These results support the hypothesis that sow farms may play a key role in the epidemiology of PRRS, at various steps along their particular production cycles. First, the introduction of replacement gilts is a first possibility of introduction of the virus to the herd. Thanawongnuwech and Suradhat [28] indicated that the sources of PRRS infection in Thailand mostly resulted from infected replacement gilts from different sources or from inadequate gilt acclimatization. In addition, risk factors associated with gilts have been reported in other risk factor studies, who found risk factors such as, for example, “not isolating gilts after purchase” [7], or “purchase of gilts from a PRRS positive herd” [29]. Second, after introduction, sows usually become involved in the mating program, where both natural mating by boar or artificial insemination can potentially transmit the virus to the sows [30]. The infection can occur through physical contact with the infected boar or through semen [3]. Upon infection, the sows can potentially transmit the disease both horizontally to other pigs on the farm, and vertically to the fetus through passage of the virus across the placenta [3]. So, the replacement of gilt, their involvement into mating programs, and their central role for feeding piglets may explain the pivotal role played by sows in terms of PRRS transmission.

Despite testing a wide set of host types, farm types and anthropogenic variables, only two variables were associated with PRRS presence at the sub-district level. Furthermore, the AUC obtained by the logistic regression was only moderate, suggesting that many other factors, probably operating at the farm level were not accounted for. The comparatively higher AUC obtained through the BRT modeling approach would need to be confirmed by external validation, because even if the AUC quantified using the test appears to be fairly high, it may still be overestimated due to spatial autocorrelation of PRRS presence/absence in the training and test set.

Further analyses, combining sub-district level factors, with factors observed at the farm level may help to clarify the respective role of risk factors identified in this study. In particular, we found no significant association between PRRS presence and the number or density of breeding boars, which was identified as a major carrier of PRRS viruses in many previous studies [7],[8],[30]. However, the role of breeding boars may be strongly influenced by the extent their movements between farms, or even, between sub-districts, and by the particular measures that are taken by the owner to prevent transmission. Moreover, the identification of several spatial clusters highlights areas where local-scale risk factors could be better investigated [31].

## Conclusions

In the long term, risk factor analysis aims to improve PRRS surveillance strategy by implementation of risk-based surveillance strategies [32]. Indeed, one can expect a more optimal use of the available resources if active surveillance is well targeted within high-risk farms or areas [32]. One approach could be, for example, to maintain passive surveillance in all types of pigs and pig farms, triggered by visible manifestation of clinical signs. To complement this, active surveillance programs could be implemented more systematically by sampling pigs in farms found to be at higher risk of PRRS infection, or suspected to play a particular role in the persistence and spread of PRRS. The results of this study suggest that farms with breeding sows may be one of those target groups, but based on previously published papers, breeding boars may be an additional and complementary focus of active surveillance due to their potential role in farm-to-farm disease transmission reported elsewhere. Simultaneously, other measures such as enhanced communication on good farm management practices and a public awareness campaign should be reinforced.

## Methods

### Data

Data on PRRS was obtained from two surveillance programs in Thailand. First, passive surveillance operates through the reporting by veterinarians of samples (organs such as lung, tonsils, and lymphnodes, and/or carcass of dead pig and/or serum of live pig) taken from pigs with clinical symptoms matching those of PRRS. Those samples are submitted to laboratories to be evaluated by RT-PCR. Second, a serological surveillance was undertaken in a set of farms with 30 sero-samples collected per flock and evaluated by ELISA. If a positive is found, new samples are taken 2–3 weeks later and evaluated with both ELISA and RT-PCR. It was not possible to differentiate between the mild PRRS and the new HP-PRRS variant by RT-PCR due to the lack of specific primers at the time when the samples were analysed. Altogether, this resulted in 764 diagnosed cases (with 198 positive cases confirmed by RT-PCR) from 381 sub-districts, 225 districts, and 61 provinces used for analyses.

The time series of daily records was analyzed in two ways. First, a linear regression was carried out with the number of daily records as a dependent variable and the day number (since the first observation day) as an explanatory variable such as to quantify the presence of an overall trend. Second, we aimed to identify the day that best divided the study period in two parts, a first period with a low average number of daily records, and another one with a high average number of daily records. So linear regressions were estimated with two explanatory variables: the day number as a continuous predictor in one hand, and the period, as a dummy variable separating observations in two groups, before and after, a given date. One linear regression per date was estimated, and the date resulting in the highest F-statistic was considered as the best date for separating observations in two groups.

For two reasons, we divided our analyses in two periods, one period running from January to July 2010, and another one from August to December 2010. First, we noted a significant increase in the number of PRRS daily records starting in late August, through the procedure described above. Second, the first identification of HP-PRRS resulted in an information campaign aiming to increase the awareness of farmers and veterinarians to this important disease, and this may have contributed to the increase in reported cases. So, there was much interest in treating those two periods separately.

The presence of spatial clusters of PRRS outbreaks in 2010 was analyzed based on the spatial scan statistic proposed by Kulldorff and Nagarwalla [33], using the centroids of sub-districts as the locations of the observations. The outbreaks were divided in two time periods, as mentioned above, in order to differentiate clusters occurring within a particular epidemic period [34]. The SaTScan version 9.3 software was implemented with the following settings for purely spatial, Bernoulli model, scan for area with high rates, 999 replications of Monte Carlo, and the maximum percentage of the population at risk to be included in a cluster of 20%.

For the development of the statistical spatial model relating PRRS data to risk factors, there were too few cases in the first period to train a model. Therefore, the analysis focused on all cases reported between August and December 2010, which were pooled and converted into presence and absence of PRRS at the sub-district level. The presence was defined as the identification of at least one pig testing positive in the sub-district, resulting in 82 sub-districts that were PRRS positive and 7,334 sub-districts that had either no samples submitted or had samples submitted that returned PRRS negative (Thai administrative units contain 4 levels composed of 76 provinces, 926 districts, 7,416 sub-districts, and 74,944 villages, with the median area of the sub-district being 45 km^2^).

The set of risk factors included variables describing human population, pig population census data for difference categories, farm types and variables describing different types of roads. Human population data for the year 2010 were obtained from the Department of Provincial Administration website (http://www.dopa.go.th). Pig population data for the year 2010 were collected through a field census conducted in January 2010 by local DLD staff, which included approximately 65,000 livestock volunteers who conducted house-to-house census surveys and submitted their data to the 926 local District Livestock Offices (DLOs). The Provincial Livestock Offices (PLOs) collected data from the DLOs and reported them through a web-based reporting system. Digital maps of roads used for our analyses were obtained from the Ministry of Transportation, and were categorized into 4 types: national roads (R1), rural roads (R2), concession roads (R3), and local roads (R4). The national roads connect regions or provinces. Rural roads are connected to national roads and allow reaching rural areas within provinces. Local roads only serve local traffic or link rural roads and local areas. Finally, concession roads serve private areas and are constructed by private contractors such as housing development or communities. The road data were aggregated at the sub-district level by their total length divided by the sub-district area. The final set of variables available in the 7,416 sub-districts of Thailand and considered in the analyses are presented in Table 4, with a total of 35 variables grouped into 8 categories including the number of pig per sub-district by type, the number of pig farms per sub-district, the pig density, the farm density, the number of pigs per farm, road density, and sub-district level human population counts. Sub-districts with no pig population census (14.5%) were located in the cities (mostly in Bangkok Metropolitan Region) and in three provinces in the south where the majority of people are Muslim. These sub-districts were excluded from the analysis resulting in a dataset having a total of 6,341 sub-districts. Several variables were included expressed in absolute numbers or as density because we had little information as to whether PRRS transmission could be a density dependent, or density independent process.

**Table 4:** Risk factors considered in the analysis of PRRS occurrences in Thailand

**Categories**	**Variables**
Number of pigs per sub-district by type	(i) number of native pigs, (ii) number of breeding boars, (iii) number of breeding sows, (iv) number of breeding piglets, and (v) number of fattening pigs
the number of pig farms per sub-district	(i) number of farms with native pigs, (ii) number of farms with breeding boars, (iii) number of farms with breeding sows, (iv) number of farms with breeding piglets, and (v) number of farms with fattening pigs.
Pig density*	(i) native pig density, (ii) breeding boar density, (iii) breeding sow density, (iv) breeding piglet density, and (v) fattening pig density
Farm density**	(i) density of farms with native pigs, (ii) density of farms with breeding boars, (iii) density of farms with breeding sows, (iv) density of farms with breeding piglets , and (v) density of farms with fattening pigs
The number of pigs per farm	(i) number of farms with less than 25 heads of pigs, (ii) number of farms with less than 50 heads of pigs, (iii) number of farms with less than 100 heads of pigs, (iv) number of farms with less than 500 heads of pigs, (v) number of farms with less than 1000 heads of pigs, and (vi) number of farms with more than 1000 heads of pigs
Farm types***	(i) number of farms with breeding boars and sows, (ii) Number of farms with breeding boars, breeding sows, and breeding piglets, (iii) number of farm with breeding boars, breeding sows, breeding piglets, and fattening pigs, and (iv) number of farms with breeding sows and breeding piglets
Road density****	(i) road1 density, (ii) road2 density, (iii) road3 density, (iv) road4 density
sub-district level human population counts	Total human population

Thirty-five explanatory variables in 8 categories included in analyses of PRRS occurrences in Thailand, estimated at the sub-district level.

*calculated by no. of pig divided by sub-district area (km^2^).

**calculated by no. of farm divided by sub-district area (km^2^).

***farms categorized by types of breeding pigs and fattening pigs (not included the native pigs).

****calculated by length of road divided by sub-district area (km^2^).

The association between the presence/absence of PRRS at the sub-district level and risk factors was analyzed using two approaches: logistic multiple regression and boosted regression tree (BRT). In logistic regression, the logit transform of the probability of the events (absence/presence) is modeled as a linear function of a set of explanatory variables [31]. It has been applied for predicting risks in many infectious disease occurrences [24]–[26] and was used here because it remains one of the most widely used and familiar techniques used in epidemiology. The BRT model approach differs from standard regression techniques by creating a single best model from a large number of relatively simple models, each being formed by a regression tree. In this way, the model combines the strengths of two algorithms, regression trees and boosting [23]. This method is increasingly used in ecological analyses in order to predict the distribution of species and has now started gaining applications in epidemiological studies [26],[35]. We considered using BRT to complement the logistic model because of its higher predictive performance reported in several studies [26],[35],[36] and its ability to fit complex non-linear relationships and interactions between risk factors [23]. Both analyses were carried out using R, and the BRT model was implemented from the set of functions provided by Elith *et al*. [23] (an extension to Ridgeway’s “gbm” library by allowing to find an optimum number of trees through cross-validation).

Multivariate logistic regression models were preceded by univariate logistic regressions used to screen all variables, keeping only those variables associated with the outcome of a *p-value* < = 0.1. All variables found significant at the level alpha = 0.1 were entered in a multivariate logistic regression model, and removed one by one, starting with the variable showing the lowest contribution to the model prediction (lowest change in log-likelihood upon removal). This procedure continued until all variables in the model were significant at the alpha level of 0.05. The method recently proposed by Crase *et al*. [37] was used to account for spatial autocorrelation in both the univariate and multivariate logistic regression models. Spatial autocorrelation, which is the tendency of neighboring points to be more similar than those distant apart, is an important bias to the assumption of independence between logistic regression residuals [38]. Augustin *et al*. [39] proposed an autologistic regression approach to account for spatial autocorrelation by including an autocovariate (autoregressive term) in the model, where the autoregressive term is estimated by averaging the residuals weighted by their distance among a set of neighbors defined by the limit of spatial autocorrelation. The method proposed by Crase *et al*. [37], is an extension of the autologistic regression approach proposed by Augustin *et al*. [39] and was found to be more successful at removing spatial autocorrelation in models residuals [25]. The approach consists in a three-step process. First, a logistic regression model is run with all risk factors and a spatial correlogram of the model residuals is estimated to identify the range of spatial autocorrelation. Second, an autoregressive term is estimated as the local mean of the first model residuals within a radius corresponding to the spatial autocorrelation range. Third, a second multiple logistic regression model including this autoregressive term as covariate is fitted.

Both the autologistic and BRT models were subject to bootstrapping of the analyses over 100 repetitions. The main purpose of the bootstrapping was twofold. First, there was a very low proportion of positives in our data set (78 out of 6,341), which can introduce bias into the logistic regression analysis [25],[39]. Second, the bootstrapping also aimed to prevent over-fitting, i.e. modelling the noise rather than the main pattern in the data by ensembling through a population of models trained with different subsets of data. So, nine times the number of positive was randomly selected at each bootstrap in order to maintain 10% of the positive values of the outcome variable. This 10% ratio was chosen because previous studies found that logistic regression tended to be biased when the prevalence in the dependent variable was lower than 10% [40]. This set was then divided in two parts: a model set used to train the model and containing 60% of the presence and absence points, and a test set containing 40% of the points and used to quantify the goodness of fit.

We proceeded in three steps to develop the BRT model. First, we identified BRT parameters (learning rates, number of trees per step, tree complexity, bag fraction) that provided a reasonably quick reduction in deviance, in a model using only human population density as a predictor. With this set of parameters, a forward entry variable selection procedure was implemented where each variable was tested in turn, in addition to human population, producing a model with two predictors. The average AUC over 100 bootstraps of each model was estimated, and the model resulting in the highest AUC in the test set was selected. When compared with the conventional regression models, BRT has no *p-values* to indicate the significance of individual predictors [23]. We decided to select variables according to their capacity to increase the classification power measured by the area under the curve (AUC) of the receiver operating characteristics (ROC) plots. AUC is a quantitative measure of the overall fit of the model that varies from 0.5 (chance event) to 1.0 (perfect fit) [41]. Although AUC was recently criticized as an absolute measure of goodness of fit by many authors, it remains valuable in comparing the performances of several models tested on the same data set. We then tested a three-predictor model where a third predictor was tested in addition to the two-predictor model identified in the previous step. Here again, the improvement was quantified using the average AUC evaluated on the test set. Each of the variables was added to the model, the AUC of each running was measured. This forward-entry procedure was carried out until the average AUC reached a maximum.

Parameters and outputs from the 100 bootstrapped models were averaged for tables and graphs outputs. For the autologistic multiple regression models, we estimated the mean and standard deviation of the coefficients, the average change in log-likelihood upon removal (ChLL), the mean and 95% confidence interval of adjusted odds ratio of each variable with the autoregressive term, and the mean AUC of the models (without the autoregressive term to avoid artificially inflating the level of predictability). For the BRT models, we estimated the mean and standard deviation of the relative contributions made by each risk factor to the models and the mean AUC of the models. In the BRT model, the relative contribution of a predictor is estimated from the number of times a variable was selected for splitting regression trees weighted by the improvement of the model produced by that split [42]. The predicted values from both modeling approaches for all sub-districts of Thailand were averaged and mapped.

### Ethical considerations

This study was approved by the Department of Livestock Development, Thailand.

## Competing interests

The authors declare that they have no competing interests.

## Authors’ contributions

WT and MG conceived and designed the study. WT generated the raw data and performed statistical analysis with contributions from MG and CL. WT drafted the paper, which MG and CL critically reviewed and revised. SK and TN provided raw data. All authors read and approved the final manuscript.
